# Sparse CNT networks with implanted AgAu nanoparticles: A novel memristor with short-term memory bordering between diffusive and bipolar switching

**DOI:** 10.1371/journal.pone.0264846

**Published:** 2022-03-31

**Authors:** Maik-Ivo Terasa, Pia Holtz, Niko Carstens, Sören Kaps, Franz Faupel, Alexander Vahl, Rainer Adelung

**Affiliations:** 1 Chair for Functional Nanomaterials, Institute for Materials Science, Faculty of Engineering, Kiel University, Kiel, Germany; 2 Chair for Multicomponent Materials, Institute for Materials Science, Faculty of Engineering, Kiel University, Kiel, Germany; National University of Ireland, Galway, IRELAND

## Abstract

With this work we introduce a novel memristor in a lateral geometry whose resistive switching behaviour unifies the capabilities of bipolar switching with decelerated diffusive switching showing a biologically plausible short-term memory. A new fabrication route is presented for achieving lateral nano-scaled distances by depositing a sparse network of carbon nanotubes (CNTs) via spin-coating of a custom-made CNT dispersion. Electrochemical metallization-type (ECM) resistive switching is obtained by implanting AgAu nanoparticles with a Haberland-type gas aggregation cluster source into the nanogaps between the CNTs and shows a hybrid behaviour of both diffusive and bipolar switching. The resistance state resets to a high resistive state (HRS) either if the voltage is removed with a retention time in the second- to sub-minute scale (diffusive) or by applying a reverse voltage (bipolar). Furthermore, the retention time is positively correlated to the duration of the *Set* voltage pulse. The potential for low-voltage operation makes this approach a promising candidate for short-term memory applications in neuromorphic circuits. In addition, the lateral fabrication approach opens the pathway towards integrating sensor-functionality and offers a general starting point for the scalable fabrication of nanoscaled devices.

## Introduction

After the memristor’s postulation by Chua *et al*. [1] and the reported link between memristor theory and resistive switching in TiO_2_ thin films by Strukov *et al*. in 2008 [2], the potential of resistive switching phenomena has led to a broad variety of research directions. The application potential ranges from non-volatile memory [3] over bio-inspired neural networks as a promising approach to overcome the von-Neumann bottleneck [4] to the concept of a “memsensor” joining memristive with sensitive functionality, allowing for unique features such as habituation to a permanent background signal [5].

Different types of memristive devices based on their switching mechanisms have been reported such as valence change mechanism (VCM), phase change materials (PCM) or electrochemical metallization (ECM), with different characteristics e.g. bipolar, unipolar or diffusive switching [6–12]. ECM cells commonly consist of a dielectric layer of a few nm in thickness between two metal electrodes, where one is electrochemically active e.g. Cu or Ag [9, 13]. Field-driven oxidation and motion of metal ions as well as their subsequent reduction at the cathode lead to the formation of a metal filament switching the device resistance from matrix determined to metal determined. Due to their bipolar switching behaviour ECM cells have been commonly discussed as candidates for non-volatile memory applications [14]. However, recent advances in ECM systems included introducing metal nanoparticles into the dielectric matrix between inert electrodes to act as an ion reservoir under exploitation of the inherent local field enhancement of nanoparticles [15], with stable diffusive switching properties that have been reported for AgAu and AgPt nanoparticles (NP) embedded in a SiO_2_ matrix in [16].

These and most other memristive devices are based on vertical stacks of thin films to achieve the nanometer scaled distances necessary for resistive switching phenomena to occur [6, 17–28]. Whereas designing memristive components in a lateral geometry makes the active interfaces on one hand accessible for investigation by surface sensitive or imaging methods such as electron microscopy and on the other hand allows them to be reached by external stimuli e.g. for surface plasmon resonance excitation [29–31] or for opening the path towards integrating sensor-features into memristive systems. However, while the layer thickness in a vertical sandwich structure can be precisely controlled by well-established deposition methods, obtaining nanoscaled distances laterally requires sophisticated and time-consuming techniques like electron beam lithography or focused ion beam deposition [10, 32].

**Fig 1:**
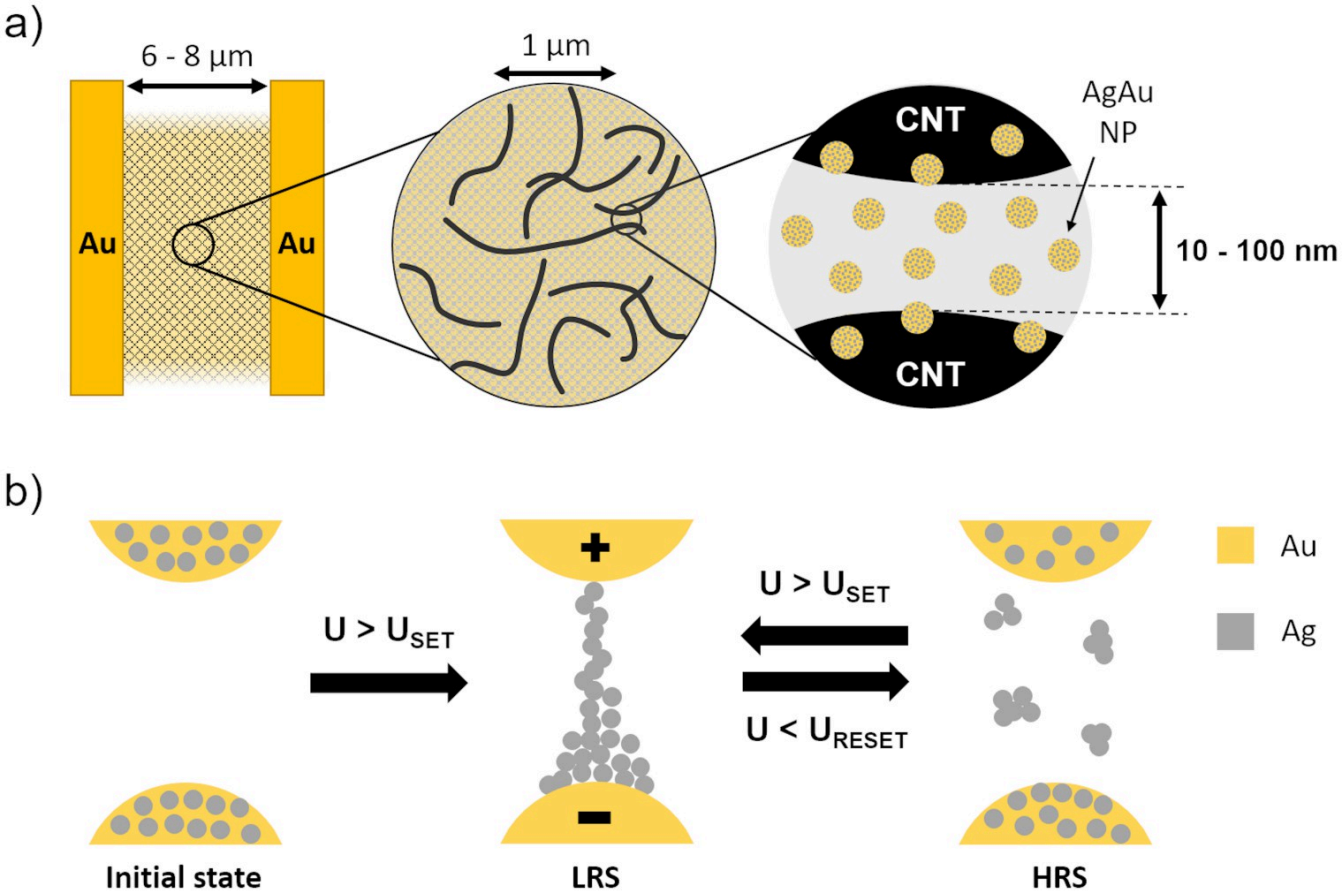
Schematic illustration of the key features and switching mechanism of the CNT/AgAu. a) Vital components of a CNT/AgAu network from left to right: The inert electrodes, the sparse CNT network and the AgAu nanoparticles inside a nanogap between two individual CNTs. b) The switching mechanism between two NPs when exposed to a potential U. LRS = Low resistive state, HRS = High resistive state.

In the scope of this work, we present a facile and scalable fabrication route for sparse CNT networks implanted with AgAu nanoparticles (in the following termed CNT/AgAu networks) in a lateral geometry, reaching the nanometer scale required for resistive switching by a combination of three length scales, as indicated in Fig 1a: Electrodes are fabricated with standard ultraviolet (UV) lithography to provide a spacing in the micrometer range (6–8 μm). The sparse CNT network provides gaps between the CNTs of up to a few hundreds of nanometers. The fabrication process uses a custom-made CNT dispersion circumventing the detrimental effects of the additives of commercially available CNT dispersions as well as allowing for a quick spin coating deposition method by using a volatile solvent. Finally, the AgAu nanoparticles, sputter deposited with a gas aggregation source (GAS) [33], yield spacings that reach the lower nanometer range. The nanoparticles implanted into gaps provided by the CNTs act as silver ion reservoirs for ECM-type switching, as illustrated in Fig 1b. The switching behaviour is a hybrid of diffusive switching with a retention time in the second- to sub-minute-scale and bipolar switching as it is possible to reset to the high resistive state (HRS) by applying reverse voltages. The retention time of a memristive system describes the time it is able to retain its resistive state, most commonly the low resistive state (LRS) [34]. For non-volatile memory applications the retention time is required to be as high as possible to prevent data loss. However, the CNT/AgAu networks with their short retention time are useful for implementing a “short-term-memory” in neuromorphic circuits. Short-term memories are efficient for storing information that becomes deprecated quickly, as the information does not have to be removed explicitly, and save power by automatically returning to a HRS. Additionally, the capability of the CNT/AgAu networks for explicit reset retains the flexibility of a traditional bipolar memory cell.

In the following sections of this work, first the fabrication route for the CNT/AgAu networks is presented. The challenges and applied methods at each fabrication step are discussed starting with the custom-made CNT dispersion. The discussion of the nanoparticle deposition includes the in-operando percolation measurement showing the sequential usage of three length scales to obtain the nanoscaled distances necessary for resistive switching. Afterwards, the results of the morphological characterization by means of scanning electron microscopy (SEM) are presented, revealing, that the fabrication route yielded an underpercolated network of CNTs and nanoparticles. In the following, the electrical characterizations are presented consisting of three different measurement modes:

- Current-voltage cycles showing the distinct high resistive and low resistive states.
- Current-voltage cycles into reverse voltage regimes showing the capability for voltage induced reset.
- Time-resolved current measurements showing the short-term memory effect of the decelerated diffusive switching behaviour and a positive correlation of the retention time to the duration of the *Set* voltage pulse.

Additionally, the data indicates that the CNT network acts as an integrated serial resistance limiting the current flow to the nA to μA regime without additional external circuitry [16] while also potentially being operable at low-voltages, resulting in a low power consumption. Finally, the proposed switching mechanism and retention are discussed with respect to filament formation and lifetime.

## Results and discussion

Most resistive switching phenomena require nanometer scaled distances due to the resulting strong electrical fields acting as a driving force for the respective switching mechanism [17, 27, 28]. While there are reliable and scalable methods to achieve these distances in vertical orientation by the deposition of thin films, the available methods to obtain this in a lateral orientation are time-consuming and inscalable [10, 32]. We developed a new method for obtaining nanometer scaled gaps with a network of CNTs deposited on a substrate with patterned gold electrodes. This CNT network must meet certain requirements:

- The network must fill the space between the electrodes.
- The CNTs must be finely dispersed, so that there are no dense agglomerations of CNTs.
- The network must be just below the percolation point, so that the distance between individual tubes is in the nanometer range.
- The CNTs must not be heavily coated by surfactants or other additives, as that would inhibit to remove short-circuiting paths by Joule heating.

For the deposition of CNT networks a custom made CNT dispersion has been prepared by mixing the following ingredients:

- Pristine CNTs as dry powder, so that they are not coated with additives initially
- Ethanol as a fast evaporating solvent to facilitate a quick sequential application of dispersion droplets onto the substrate during spin coating
- Poly(3,4-ethylenedioxythiopene) polystyrene sulfonate (PEDOT:PSS) as an additive, keeping the CNTs finely dispersed [35]

The mixture has been sonicated with an ultrasonicator to break up the CNT particles and disperse them. The PEDOT:PSS prevents re-agglomeration without impeding the resistive heating step.

A thin film of dispersion has been deposited onto the substrates via spin coating. The dispersion was dropped onto the substrate sequentially dropwise during spinning, where each drop has been left to evaporate before applying the next one, allowing for precise control over the particle density.

After deposition of a CNT network a voltage ramp has been applied to it to remove any continuous CNT paths short-circuiting the electrodes by resistive heating, which has been indicated by a sudden drop in the current readout (see S1 Fig).

The AgAu nanoparticles have been deposited by direct current (DC) magnetron sputtering using a Haberland-type gas aggregation source (GAS) identical to the one reported in [36], with a AgAu target as in [33] attached to the DC planar magnetron source. This deposition method enables precise control over particle composition and density without affecting the CNT network on the substrate [33].

**Fig 2:**
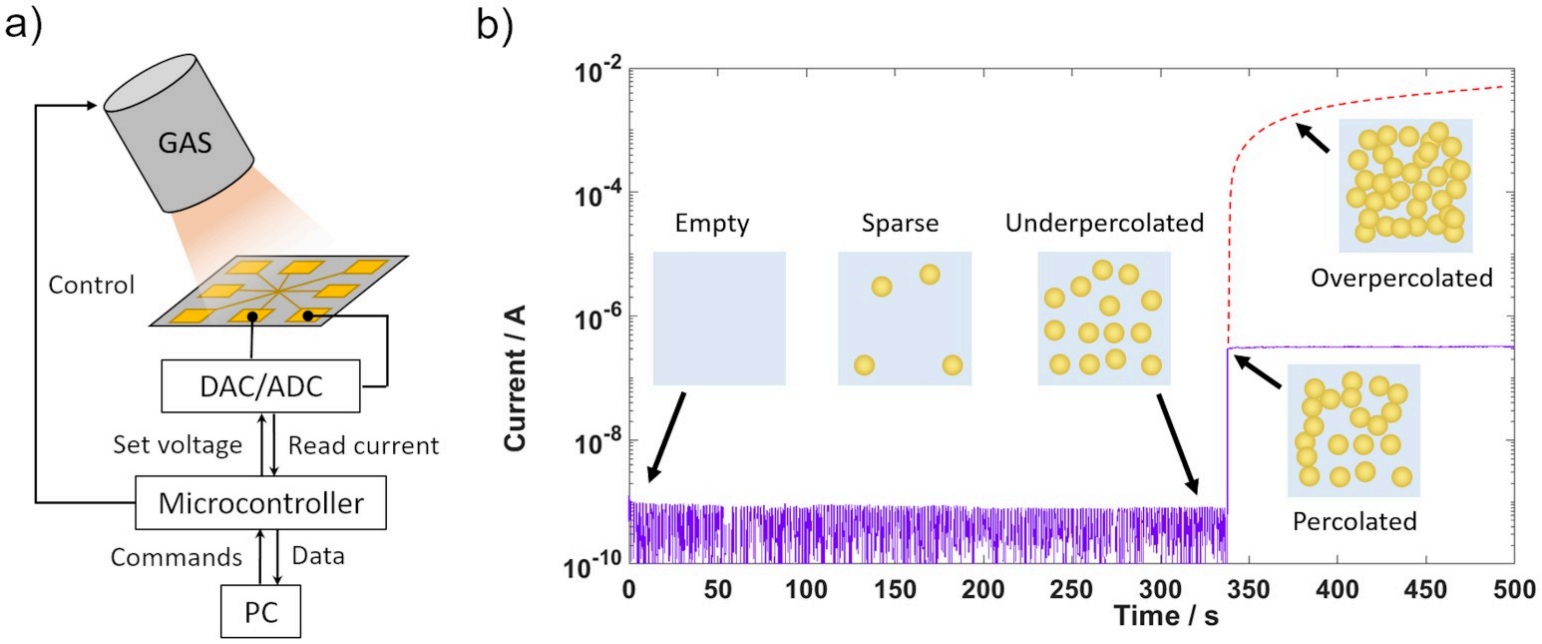
Percolation measurement for AgAu nanoparticle deposition. a) Schematic of the in-operando percolation measurement setup. b) Time-resolved current measurement across adjacent electrodes at a voltage of 3V. The time where the flowing current shows a significant increase is taken as the percolation time (337 s). The deposition has been stopped at the percolation point. The red dashed line indicates the progression of the current, if the deposition had continued, leading to an overpercolated layer of nanoparticles.

The deposition time for the nanoparticles has been set to stay below the percolation point [37]. The deposition time for the percolation point has been determined by performing electrical measurements in-operando during deposition. A schematic of the setup and the percolation measurement of a substrate with deposited CNT network is shown in Fig 2. After deposition of AgAu nanoparticles, a thin layer of SiN has been deposited on top as a protective layer without breaking vacuum. Experimental details about any step of the procedure can be found in the section “Materials & Methods”.

**Fig 3:**
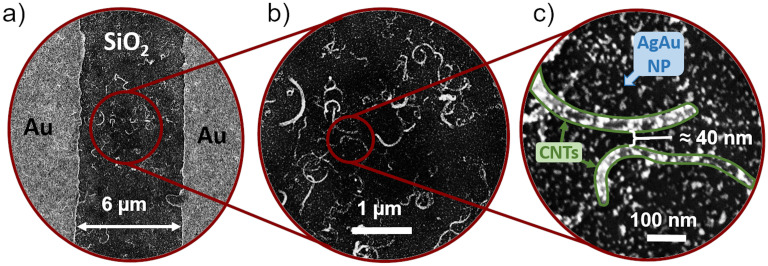
SEM micrographs of a finished [CNT/AgAu network] without SiN layer. a+b) Homogeneous sparse CNT network between the electrodes. c) A nanogap between two CNTs with deposited AgAu NP. The samples shown in the images have not been coated with SiN.

Fig 3 shows a sequence of SEM images of a CNT/AgAu network revealing the homogeneous distribution and sparseness of the CNTs. A substantial fraction of CNTs have been broken into smaller pieces during the ultrasonication step, which we assume to provide two advantages for the fabrication process: Firstly, an alleviation of entanglement and agglomeration of CNTs and secondly an increased sparseness of the network providing more gaps between CNTs and preventing individual tubes from bridging the whole network.

Fig 3c shows a CNT network with deposited underpercolated AgAu nanoparticles. The particle distribution shows spacing in the lower nanometer range enabling ECM-type memristive switching between nanoparticles [16]. Samples solely prepared with AgAu nanoparticles with an equal filling factor showed no switching behaviour in the considered voltage regime (see S2 Fig) verifying that the sparse network of CNTs fulfils its expected functionality of providing suitable nanogaps to enable the resistive switching of the nanoparticles.

**Fig 4:**
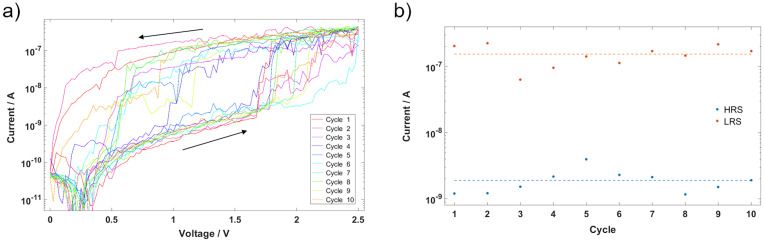
Resistive switching behaviour. a) Cyclic voltage pattern, showing resistive switching behaviour and an ON/OFF ratio of around 81 at a Read voltage of 1.5 V. b) HRS and LRS currents at Read voltage of 1.5 V for each cycle.

For CNT/AgAu networks, that did not show resistive switching below 10 V, an electric preforming step has been performed by cycling to a voltage of ± 20 V (0 V -> 20 V -> -20 V -> 0 V) over several cycles (see S3 Fig). A stable HRS corresponding to Fig 1b is reached during the second cycle, from where on the resistive switching occurs. This indicates that nanoparticle gaps in the conduction path become persistent conductive elements by forming stable filaments that are not collapsing, when the electrical field is removed. This yields lower switching voltages by decreasing the number of gaps over which the overall voltage drops. After the preforming procedure, when cycled in low voltage regimes, operation becomes stable as shown in Fig 4.

Fig 4a shows a cyclic IV-measurement of a CNT/AgAu network with an 8 μm spacing between electrodes. The voltage has been cycled from 0 V to 2.5 V and back to 0 V at a rate of 250 mV/s over ten cycles, where after each cycle the voltage has been held at 0 V for 5 s. The current response shows a clear distinction of two resistive states (see Fig 4b) and stable operation across the ten cycles with the HRS current being 1.9 ± 0.8 nA and the LRS current being 155 ± 55 nA yielding a current ratio between HRS and LRS of ≈ 81 at a *Read* voltage of 1.5 V. The data indicates the potential for low-voltage operation and shows that the network itself acts as a series resistance limiting the current flow and thus ensuring low power consumption without additional circuitry (cf. [16]).

**Fig 5:**
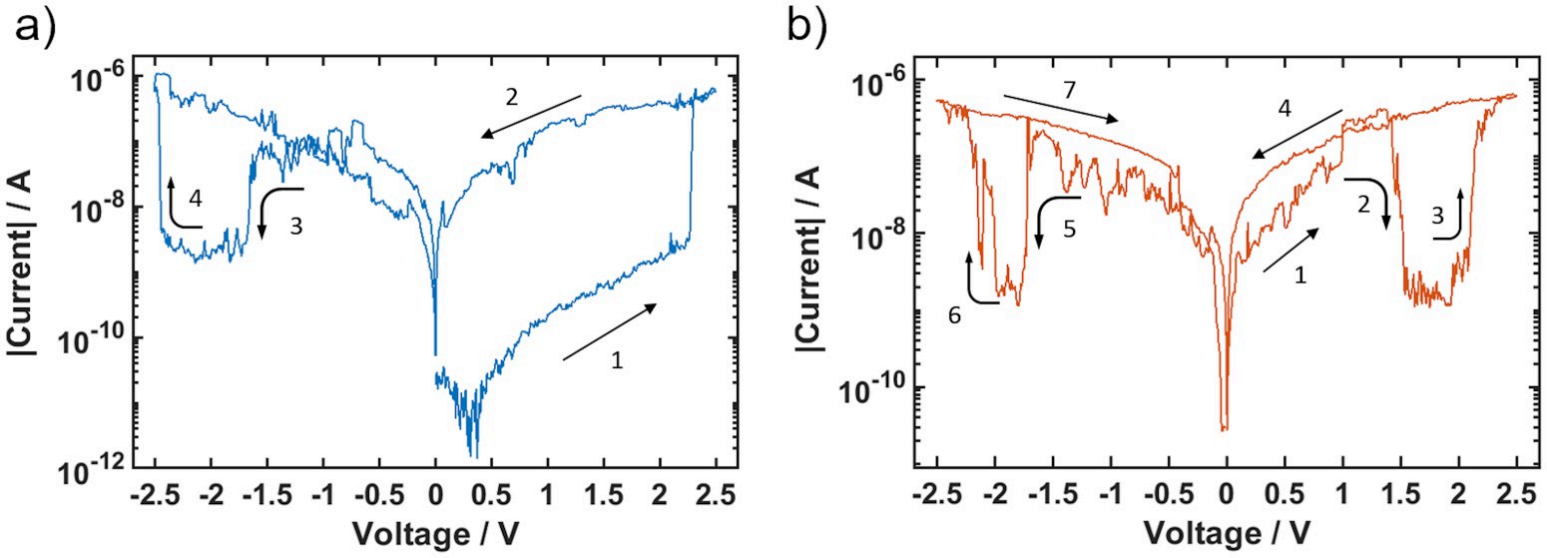
Reset behaviour with reverse voltage. a) Cycle starting in HRS. The LRS is retained until -1.5 V is applied. b) Subsequent cycle starting in LRS. The reset behaviour is symmetrical in the positive and negative voltage range. The numbers indicate in which order the resistive switching occurred.

Fig 5 shows two subsequent cycles of a CNT/AgAu network, recorded at a ramp speed of 25 mV/s. The cycle shown in Fig 5a starts in an HRS, switching into the LRS and retaining it, until a negative voltage of -1.5 V is applied. The CNT/AgAu network then switches into its LRS again upon reaching its *Set* voltage of 2.5 V. The subsequent cycle seen in Fig 5b starts in the LRS and shows that this effect is symmetrical. Reducing the ramp speed of 10 mV/s did not change the reset voltage, indicating that the effect is not accountable to the diffusive reset behaviour. These measurements indicate that, while the CNT/AgAu network also returns to its HRS over time without the application of a voltage like a diffusive device, it shows the ability of bipolar switching i.e. to be reset from LRS to HRS by reverse voltages.

**Fig 6:**
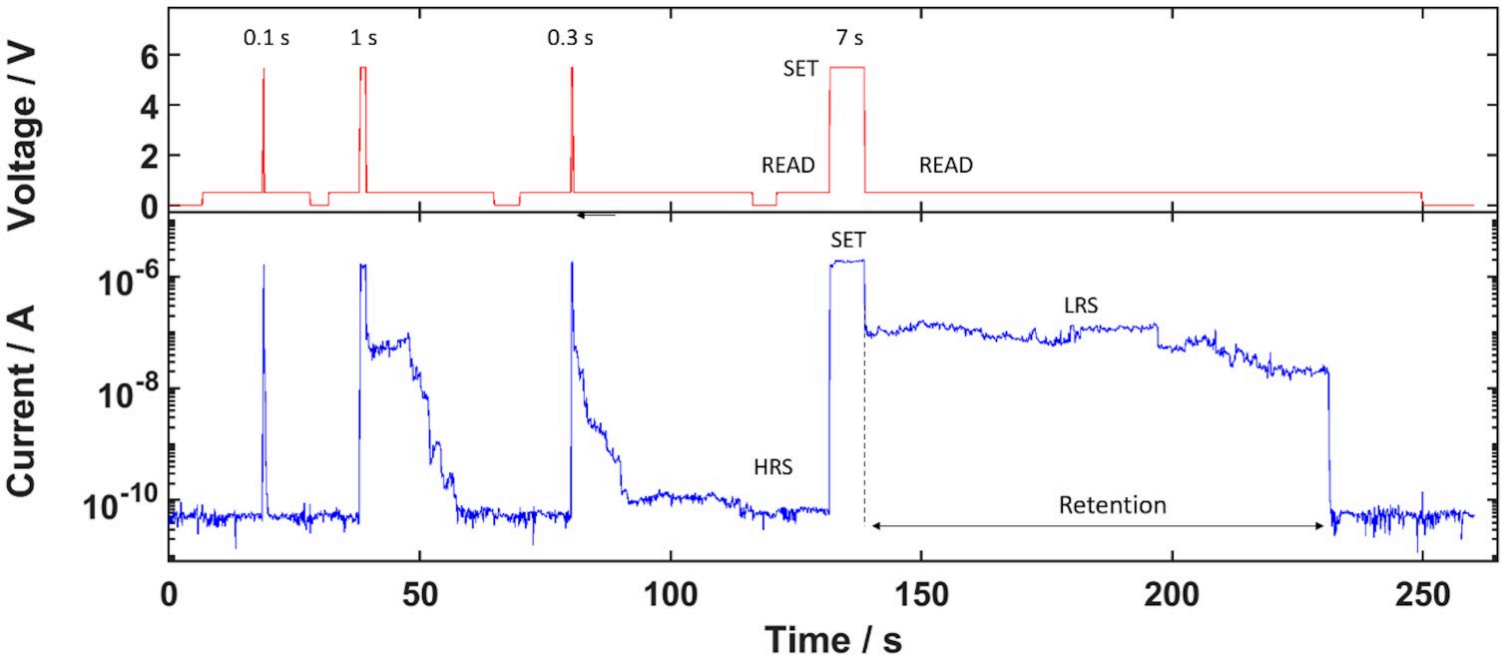
Time-resolved current measurements showcasing the retention. The upper graph shows the applied voltage pattern: Read voltage = 0.5 V, Set voltage = 5.5 V. The retention time is taken as the time from returning from Set to Read voltage to when the current reaches the HRS current. The numeric values indicate the duration of the Set voltage pulse. The Set time and retention time show a positive correlation, which can be found in the (see S4 Fig).

The time-dependent retention has been investigated by time resolved current measurements with the voltage pattern shown in the upper graph of Fig 6. The measurement starts at 0 V for several seconds to serve as a reference. The current readout at the *Read* voltage of 0.5 V before the first *Set* pulse shows that the CNT/AgAu network is in its HRS initially. After each *Set* pulse, each with different pulse durations, the current readouts at the subsequent *Read* voltage indicate a switch of the CNT/AgAu network into the LRS. The *Read* voltage has been held until it returned into its HRS, where the time from returning to the *Read* voltage until the current returned to the HRS regime has been taken as the retention time (see Fig 6). The series of pulse durations for the *Set* pulses indicates a positive correlation between the time in the *Set* state and the resulting retention time of the CNT/AgAu network i.e. a longer *Set* pulse results in a longer retention time (see also S4 Fig).

In ECM devices the resistive states originate from metal filaments formed by movement and reduction of metal ions in the electrical field [12, 16, 38, 39]. With a formed filament the device is in its LRS. When the filament ruptures, it returns to its HRS, which is due to surface tension of the filament as a restoring force, making it energetically favourable to form spherical particles depending on the thickness of the filament and the surrounding matrix material [7, 40]. In bipolar memristive ECM devices the electrodes provide a metal reservoir large enough to form stable filaments with a retention of several years [40]. The CNT/AgAu networks use the AgAu nanoparticles as metal ion reservoirs limiting the amount of silver atoms available for filament formation. Singular or few nanoparticles have been reported to show no retention due to the formed filament being thin enough to immediately collapse as soon as the electrical field as a driving force is removed [16]. In this work, however, the density of deposited AgAu nanoparticles in the CNT networks’ nanogaps yield a suitable silver reservoir to provide a substantial amount of silver ions for filament formation. The resulting filament in the gaps, where resistive switching occurs, is thick enough to be stable, so that the rupture does not happen right away without electrical field, but instead is delayed until diffusion thinned down the filament enough to collapse by surface tension.

At the same time though the amount of silver atoms is still limited such that the filament formed by an electrical field is not outright long-term stable, like in a typical bipolar device. Instead the filament formed initially is sufficiently thin to show diffusive switching. However, with prolonged application of the electrical field, the filament grows by material diffusion from other nearby nanoparticles. The longer the *Set* voltage is applied, the thicker the filament becomes. Thus, the retention time increases i.e. the time it takes for the filament to collapse after removing the electrical field.

As long as the filament holds, a reverse voltage excitation is able to break the filament. In typical bipolar ECM type systems the electrode materials are asymmetrical, so that only one voltage polarity yields a filament while the reverse polarity leads to its dissolution [12, 14, 38]. As seen in Fig 5b however, it is evident, that the behaviour of the CNT/AgAu network is symmetrical. The reset mechanism is presumed to be based on drift of the filament’s silver towards the cathode until the filament is sufficiently thinned down on the anode side to collapse, switching the CNT/AgAu network into its HRS.

## Conclusion

In this work sparse CNT networks as a new approach for obtaining nanoscaled distances in a lateral geometry as well as those networks with implanted AgAu nanoparticles as a novel lateral memristor with short-term memory capabilities and a hybrid switching behaviour between diffusive and bipolar switching have been presented.

The sparse CNT networks have shown to offer a general starting point for introducing nanoscaled gaps into laterally oriented systems. The CNTs exhibit the function of bridging substantial distances between the electrodes while gaps between individual CNTs have shown to be in the nanometer range. AgAu nanoparticles implanted into this CNT network showed ECM-type resistive switching. The switching behaviour is based on providing a substantial but still limited reservoir of silver by deposited AgAu nanoparticles, yielding a diffusive switching behaviour with a second-scale retention as well as the ability of bipolar devices to reset to the HRS by reverse voltages. It has been shown that the “memory span”, i.e. the retention, is positively correlated to the width of the *Set* voltage pulse since the retention is prescribed by the filament’s lifetime and thus its thickness. Also, it has been shown that the CNT/AgAu networks are able to reach switching voltages in the range providable by chips fabricated with complementary metal-oxide semiconductor (CMOS) technology, while the network provides an integrated serial resistance limiting the current to the nA to μA range. This makes CNT/AgAu networks a promising approach for low-power short-term memory components in neuromorphic circuits, though for a deeper understanding further investigations with respect to the detailed correlation of *Set* pulse width and retention time as well as the impact of the network topology are necessary.

## Materials & methods

The substrates were produced from commercially available Si wafers with a 500 nm thick oxide layer on top. The metal contacts have been deposited by a standard UV-lithography with a Süss Microtech MA6/BA6 mask aligner, followed by a sputtering and lift-off process. The sputter process was a DC magnetron sputter deposition of Cr with a thickness of 10 nm as an adhesive layer and on top Au with a thickness of 200 nm as a contact layer. The lift-off has been performed in an ultrasonicated acetone bath held at 40°C. After the lift-off the wafer has been cleaned and dried after which it has been cut into 11 x 11 mm pieces with a DAD3350 automated dicing saw.

The CNT dispersions have been fabricated by mixing 10 g of ethanol (99.7% purity, provided by Carl Roth) with 40 μg pristine multi-walled carbon nanotubes (MWCNT, Baytubes C150P) and 1 μl of 1.3wt% aqueous solution of PEDOT:PSS (Ossila PH1000). The ultrasonication was performed with a Sonics Vibra-Cell VC 505 with 500 W at 70% amplitude. The dispersions have been sonicated for 15 minutes with cycles of 3 s pulsing and 3 s pause. The glass tube with the dispersion has been kept in a water bath cooled with a Peltier element during sonication.

The spin coater was a Laurell WS-650MZ-23NPPB and has been rotated at a constant rate of 1500 rpm. A total of 350 μl of dispersion has been deposited dropwise on each substrate.

For the resistive heating step the applied voltage ramp was 5 V/s up to a maximum of 30 V under a current compliance of 100 μA.

The sputter deposition of nanoparticles has been performed with a Haberland-type gas aggregation source with a AgAu multicomponent target [33]. Ar was used as a process gas at a flow of 50 sccm. The magnetron power has been set to 50 W. The shutter has been kept closed after turning on the magnetron power for 30 seconds to ensure a stable deposition rate before the sample has been exposed to the sputter source. The deposition time until the percolation point is reached has been determined to be 337 s. Samples have been sputtered for 330 s to stay below the percolation point.

A SiN layer has been deposited as a protective layer by reactive sputtering using a Si target with a nitrogen flow of 4.2 sccm and a magnetron power of 20 W. The deposition time was 7 minutes, resulting in a nominal film thickness of 21 nm.

For the IV-characterization a software-controlled Keithley 2400 Source Measure Unit has been used with gold plated spring contacts as probes placed on the electrode pads. The measurement delay between data points has been determined to be ≈100 ms.

SEM images have been recorded with a Zeiss Ultra Plus at 5 kV acceleration voltage using the in-lens detector.

## Supporting information

S1 Fig Typical current measurement for Joule heating of a CNT network. The voltage has been cycled two times. After the current dropped it stayed in the limit of detection (LOD) of the measurement device.
(TIF)

S2 Fig Current measurement of a sample without CNT network. (TIF)

S3 Fig Electric preforming of a CNT/AgAu network over four cycles. Red = positive half-cycle, Blue = negative half-cycle. a) During the first cycle there is no distinct Set behaviour. The Ag-ions are located inside the AuNP and are gradually dragged out by the electric field. b) During the positive half-cycle the CNT/AgAu network reaches the LRS illustrated in Fig 1b, so that the following cycles show corresponding ECM-type resistive switching.
(TIF)

S4 Fig Retention time vs. set pulse duration. The two points at each Set pulse duration indicate the time when the current degression starts after returning to the *Read* voltage and when the current reaches the HRS current regime.
(TIF)

S1 Data Raw data for Figs 2 and 4–6, and S1–S3 Figs. (ZIP)

S1 File Original SEM micrograph for Fig 3. (ZIP)
