# Supplementary figures and images for: Sparse CNT networks with implanted AgAu nanoparticles: A novel memristor with short-term memory bordering between diffusive and bipolar switching
Source: PLoS One. 2022 Mar 31;17(3):e0264846. doi: 10.1371/journal.pone.0264846 (PMC8970472; doi:10.1371/journal.pone.0264846)

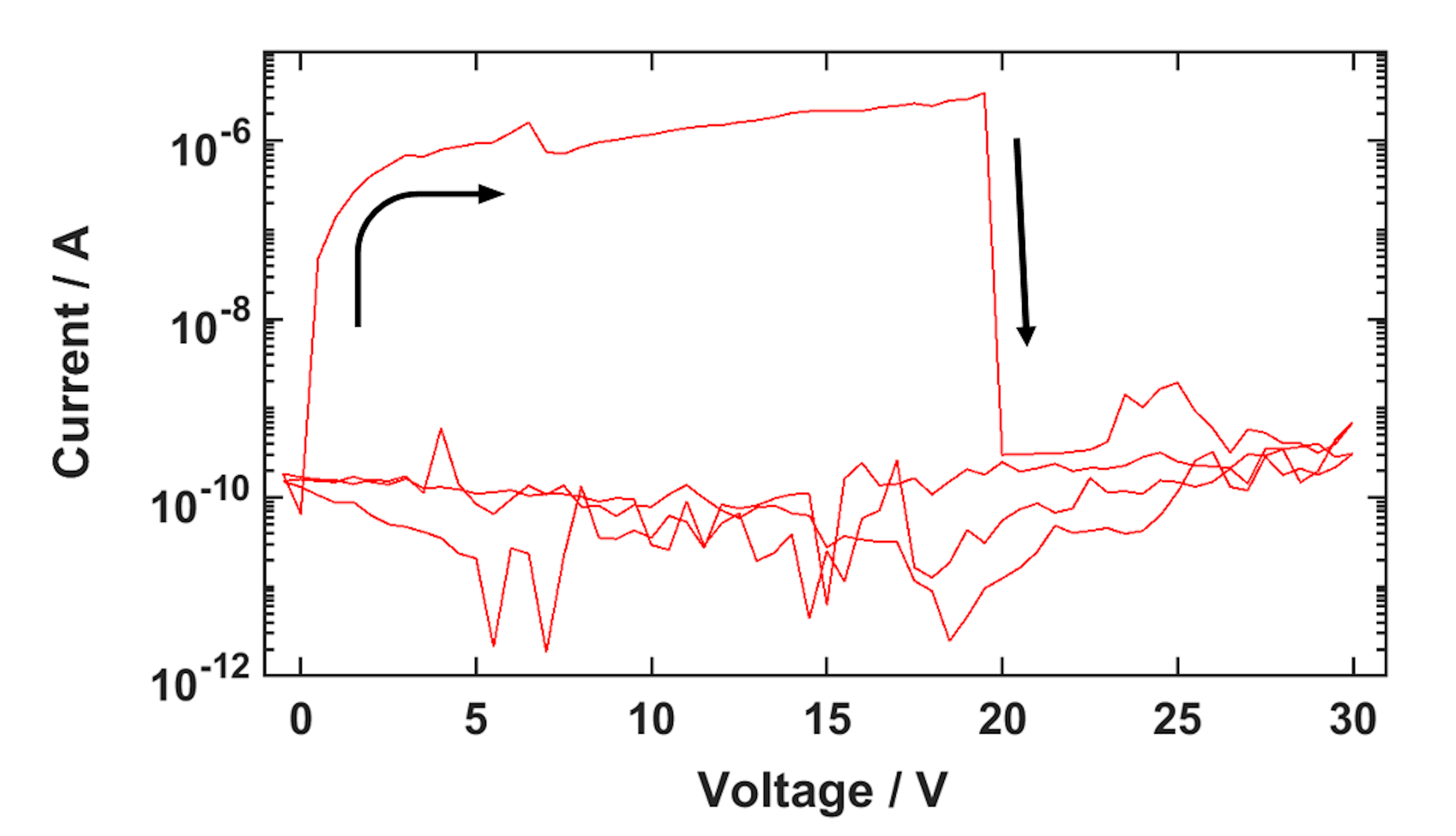

Supplement: S1 Fig — The voltage has been cycled two times. After the current dropped it stayed in the limit of detection (LOD) of the measurement device. (TIF) [file pone.0264846.s001.tif]

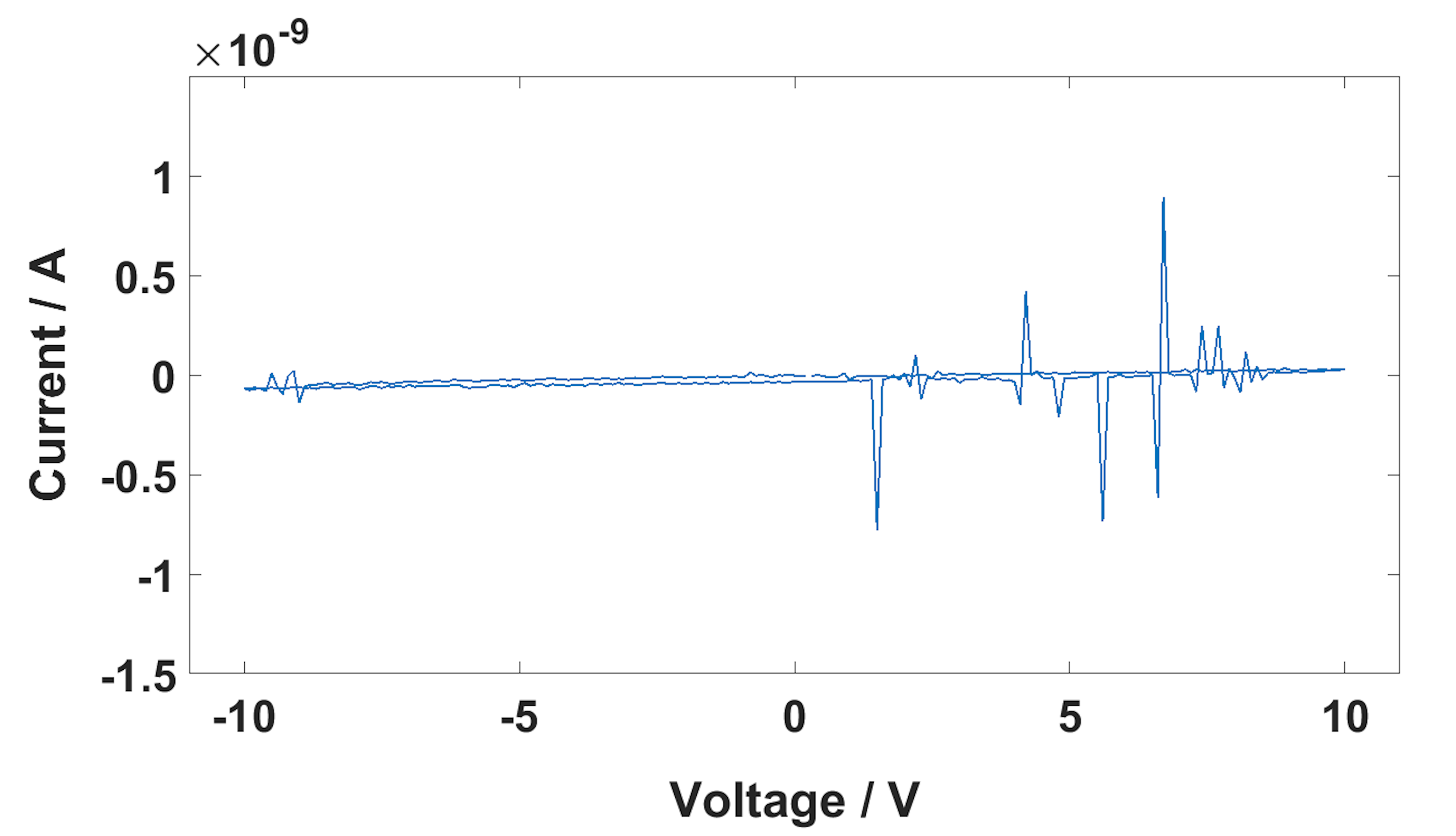

Supplement: S2 Fig — (TIF) [file pone.0264846.s002.tif]

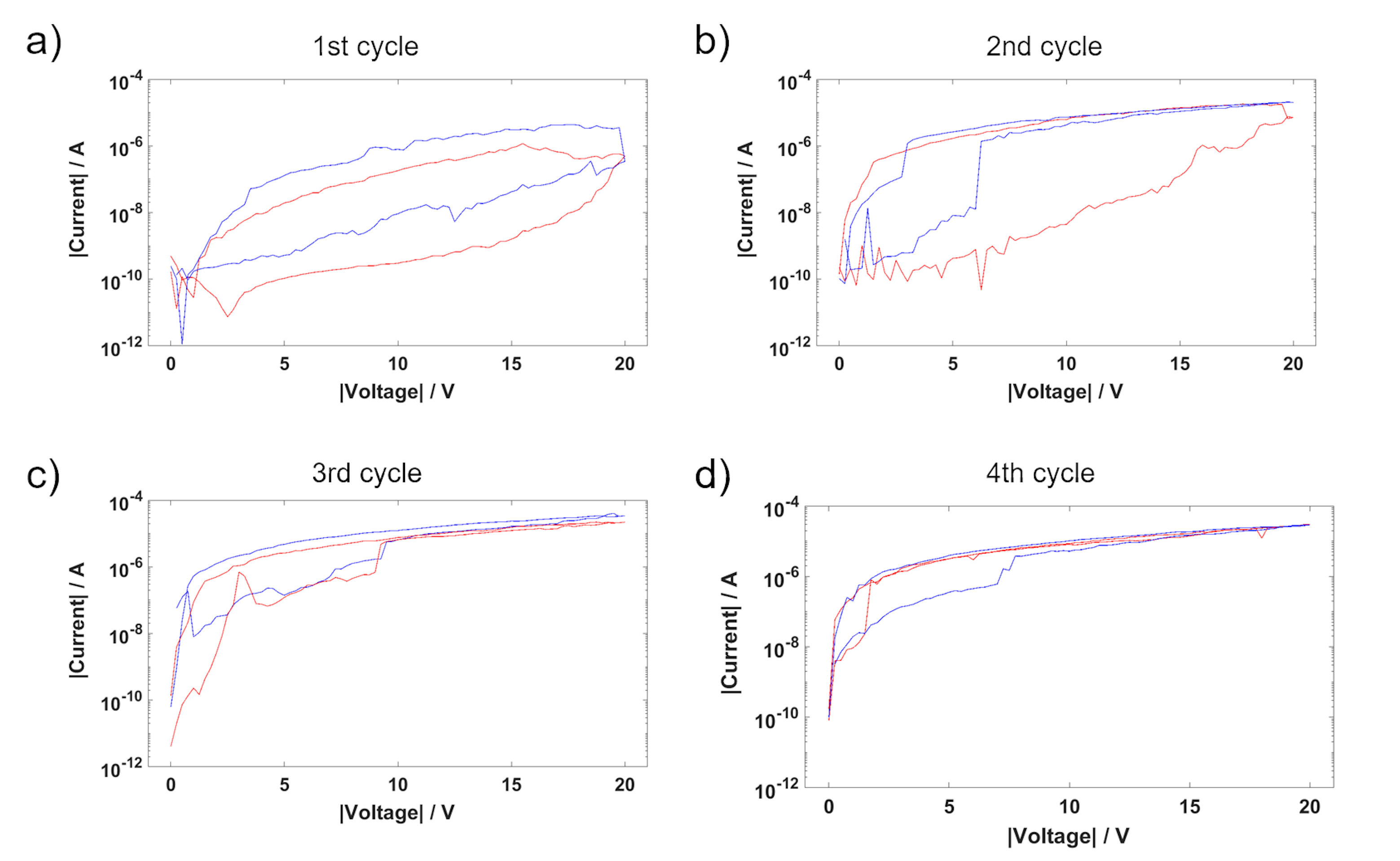

Supplement: S3 Fig — Red = positive half-cycle, Blue = negative half-cycle. a) During the first cycle there is no distinct Set behaviour. The Ag-ions are located inside the AuNP and are gradually dragged out by the electric field. b) During the positive half-cycle the CNT/AgAu network reaches the LRS illustrated in Fig 1b, so that the following cycles show corresponding ECM-type resistive switching. (TIF) [file pone.0264846.s003.tif]

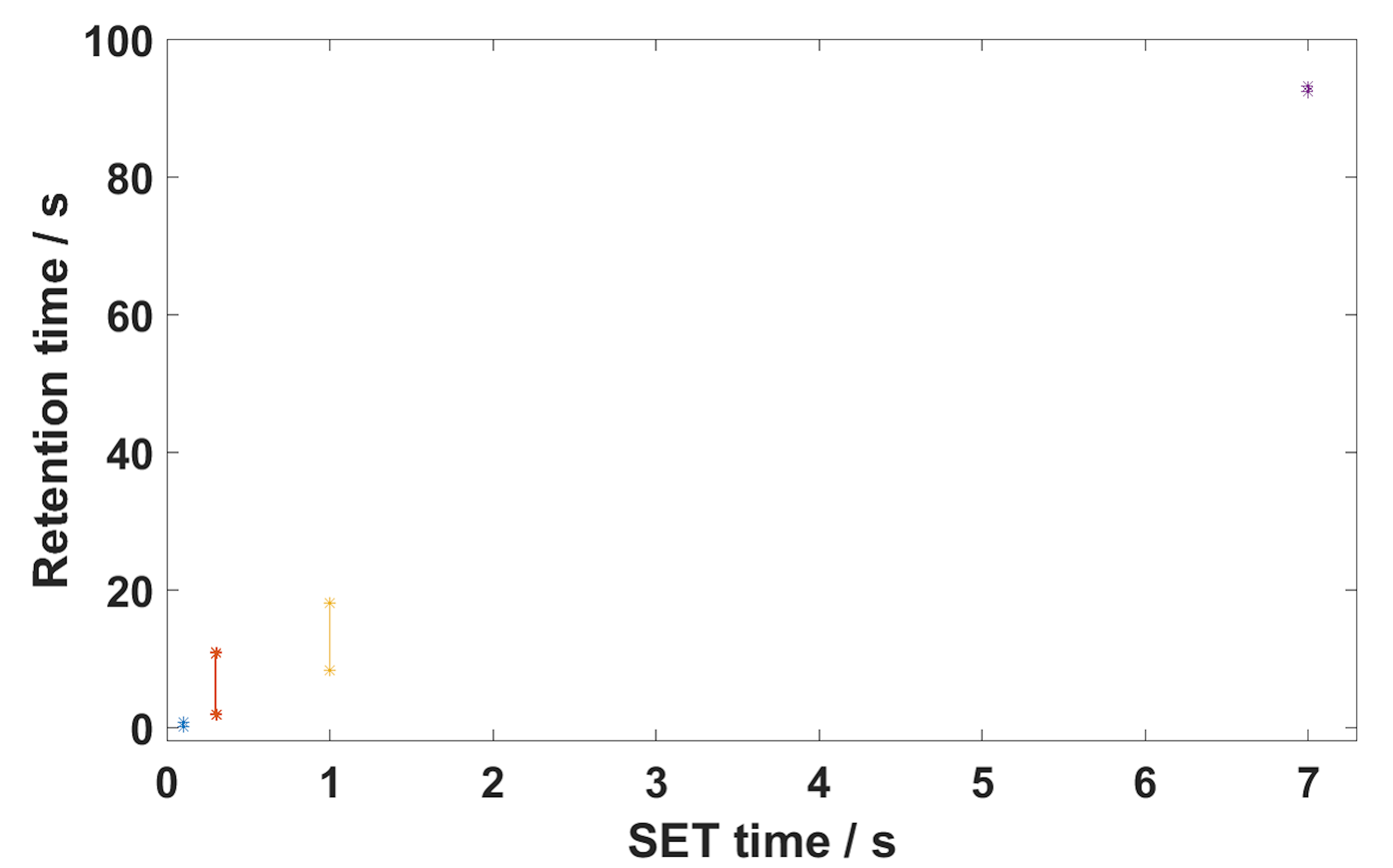

Supplement: S4 Fig — The two points at each Set pulse duration indicate the time when the current degression starts after returning to the Read voltage and when the current reaches the HRS current regime. (TIF) [file pone.0264846.s004.tif]
